# Evaluation of the GeneXpert MTB/RIF in patients with presumptive tuberculous meningitis

**DOI:** 10.1371/journal.pone.0198695

**Published:** 2018-06-18

**Authors:** Tatiana Metcalf, Jaime Soria, Silvia M. Montano, Eduardo Ticona, Carlton A. Evans, Luz Huaroto, Matthew Kasper, Eric S. Ramos, Nicanor Mori, Podjanee Jittamala, Kesinee Chotivanich, Irwin F. Chavez, Pratap Singhasivanon, Sasithon Pukrittayakamee, Joseph R. Zunt

**Affiliations:** 1 Department of Clinical Tropical Medicine, Faculty of Tropical Medicine, Mahidol University, Bangkok, Thailand; 2 Northern Pacific Fogarty Global Health Fellowship Program, National Institutes of Health, University of Washington, Seattle, Washington, United States of America; 3 Department of Tropical and Infectious Diseases, Hospital Nacional Dos de Mayo, Lima, Peru; 4 US Naval Medical Research Unit No. 6, Callao, Peru; 5 Section of Infectious Diseases & Immunity, Imperial College London, and Wellcome Trust Imperial College Centre for Global Health Research, London, United Kingdom; 6 Innovation for Health & Development (IFHAD), Laboratory of Research and Development, Universidad Peruana Cayetano Heredia, Lima, Peru; 7 Department of Tropical Hygiene (Biomedical and Health Informatics), Faculty of Tropical Medicine, Mahidol University, Bangkok, Thailand; 8 Department of Neurology, University of Washington, Seattle, Washington, United States of America; 9 Department of Global Health, University of Washington, Seattle, Washington, United States of America; 10 Department of Medicine, University of Washington, Seattle, Washington, United States of America; 11 Department of Epidemiology, University of Washington, Seattle, Washington, United States of America; St Petersburg Pasteur Institute, RUSSIAN FEDERATION

## Abstract

**Background:**

Meningitis caused by *Mycobacterium tuberculosis* is a major cause of morbidity and mortality worldwide. We evaluated the performance of cerebrospinal fluid (CSF) testing with the GeneXpert MTB/RIF assay versus traditional approaches for diagnosing tuberculosis meningitis (TBM).

**Methods:**

Patients were adults (*n =* 37) presenting with suspected TBM to the Hospital Nacional Dos de Mayo, Lima, Peru, during 12 months until 1st January 2015. Each participant had a single CSF specimen that was divided into aliquots that were concurrently tested for *M*. *tuberculosis* using GeneXpert, Ziehl-Neelsen smear and culture on solid and liquid media. Drug susceptibility testing used Mycobacteria Growth Indicator Tube (MGIT 960) and the proportions method.

**Results:**

81% (30/37) of patients received a final clinical diagnosis of TBM, of whom 63% (19/30, 95% confidence intervals, CI: 44–80%) were HIV-positive. 22% (8/37, 95%CI: 9.8–38%), of patients had definite TBM. Because definite TBM was defined by positivity in any laboratory test, all laboratory tests had 100% specificity. Considering the 30 patients who had a clinical diagnosis of TBM: diagnostic sensitivity was 23% (7/30, 95%CI: 9.9–42%) for GeneXpert and was the same for all culture results combined; considerably greater than 7% (2/30, 95%CI: 0.82–22%) for microscopy; whereas all laboratory tests had poor negative predictive values (20–23%). Considering only the 8 patients with definite TBM: diagnostic sensitivity was 88% (7/8, 95%CI: 47–100%) for GeneXpert; 75% (6/8, 95%CI: 35–97%) for MGIT culture or LJ culture; 50% (4/8, 95%CI 16–84) for Ogawa culture and 25% (2/8, 95%CI: 3.2–65%) for microscopy. GeneXpert and microscopy provided same-day results, whereas culture took 20–56 days. GeneXpert provided same-day rifampicin-susceptibility results, whereas culture-based testing took 32–71 days. 38% (3/8, 95%CI: 8.5–76%) of patients with definite TBM with data had evidence of drug-resistant TB, but 73% (22/30) of all clinically diagnosed TBM (definite, probable, and possible TBM) had no drug-susceptibility results available.

**Conclusions:**

Compared with traditional culture-based methods of CSF testing, GeneXpert had similar yield and faster results for both the detection of *M*. *tuberculosis* and drug-susceptibility testing. Including use of the GeneXpert has the capacity to improve the diagnosis of TBM cases.

## Introduction

Despite being a preventable and curable disease, tuberculosis (TB) remains in the top 10 causes of death worldwide and is the most frequent infectious cause of death [1]. The incidences of multidrug-resistant (MDR) and extensively drug-resistant (XDR) TB are increasing. These require extended treatment and are associated with higher morbidity and mortality. The risk of acquiring TB, the rate of progression from latent to active disease, as well as the morbidity and mortality associated with TB, all significantly increase with HIV co-infection [2, 3]. Furthermore, HIV co-infected patients are five times more likely to develop central nervous system (CNS) TB, which causes poor clinical outcomes including death [2, 3, 4, 5]. In 2016 approximately 10 million people developed TB worldwide, of whom an estimated 1.4 million (10%) were co-infected with HIV. An estimated 1.6 million deaths were caused by TB, 374,000 of whom were in people co-infected with HIV [6].

Tuberculous meningitis (TBM) is one of the most severe forms of TB. TBM occurs when *Mycobacterium tuberculosis (M*. *tuberculosis)* invades the membranes and fluid surrounding the brain and spinal cord. Without appropriate treatment, the disease will invariably progress, resulting in neurologic impairment, CNS damage and often death. Globally, TBM represents approximately 5% of extra-pulmonary TB (EPTB) cases. At the Hospital Nacional Dos de Mayo (HNDM) in Lima, over a 10-year period, TBM made up 29% of all EPTB cases, and 11% of all TB cases. For example, in the year 2014, 350 patients were hospitalized at HNDM with TB and 50% of these patients had extra-pulmonary TB; TBM cases made up 38% of all EPTB; TBM comprised 19% of all TB cases, according to HNDM hospital records.

Diagnosing TBM is more difficult than other forms of bacterial meningitis, partially because symptoms generally do not develop as suddenly as with classic bacterial meningitis and because TB produces a paucibacillary infection that is difficult to detect in CSF. When diagnosis via detection of *M*. *tuberculosis* in CSF is not possible, an unconfirmed diagnosis of TBM is often made based on the combination of clinical and CSF findings. Current laboratory diagnostic methods are mainly culture-dependent (using liquid and/or solid culture media) and generally take several weeks to generate a detectable bacteriological replication and concurrently or usually subsequently, a drug sensitivity profile. Conventional diagnostic techniques for MDR-TB usually involve a long and arduous process that requires a series of steps to isolate the mycobacteria in clinical samples, identify the *M*. *tuberculosis* complex, and then in vitro analysis to determine the sensitivity of the strain to anti-tuberculous drugs.

One of the greatest challenges clinicians face concerning TBM is making a rapid and accurate diagnosis, which requires prompt recognition using a combination of clinical criteria, radiological and laboratory diagnostics. Usually, initiation of empiric first-line TB treatment for suspected TBM occurs without laboratory confirmation that TBM is the correct diagnosis, and without evidence of TB drug-susceptibility.

The GeneXpert MTB/RIF (Cepheid, Sunnyvale, CA USA) is an automated closed-cartridge system used to simultaneously detect *M*. *tuberculosis* and rifampicin (RIF) resistance within 2 hours. It also assigns a semi-quantitative estimate (very low, low, medium and high) of the concentration of TB bacilli in the sample, based on the cycle threshold.

In 2013, the World Health Organization endorsed GeneXpert MTB/RIF for the diagnosis of extra-pulmonary TB in adults and children, including for CSF in patients with suspected TBM [7]. However, the recommendations were based on very low quality data [7, 8]. For these reasons, TB experts stress the need to re-evaluate the performance of GeneXpert and improve and standardize CSF sample processing to maximize sensitivity and specificity [9, 10, 11]. Furthermore, several studies have raised concern about the use of culture as the reference standard in the evaluation of the GenXpert for EPTB specimens [12, 13].

We conducted a clinical validation study to evaluate the diagnostic performance of GeneXpert MTB/RIF assay in a clinical cohort of patients with suspected TBM from an area in Peru with endemic TB and HIV infections. GeneXpert results were compared against two comparators: (1) sufficient clinical suspicion or evidence for TBM treatment to be commenced; and (2) positive *M*. *tuberculosis* CSF culture-confirmed TBM.

## Materials and methods

### Ethics statement

The study protocol was approved by the Institutional Review Boards of the U.S. Naval Medical Research Unit No. 6, Mahidol University, the Hospital Nacional Dos de Mayo (HNDM) and the University of Washington in compliance with all applicable Federal regulations governing the protection of human subjects. All patients or their guardians chose to give informed written consent prior to research participation.

### Setting and population

The study setting was the HNDM, a 600-bed national reference hospital nationally renowned for its infectious diseases and tropical medicine departments, located in Lima, Peru. The HNDM serves adult patients from the Lima province, an area of approximately 10 million inhabitants. This predominantly low-income, high-density population contains areas of high-risk HIV and TB incidence and transmission. Inclusion criteria were all adult patients ≥18 years of age presenting with a suspected diagnosis of TBM to the HNDM hospital, Lima, Peru. Exclusion criteria were inability or unwillingness to give informed written consent, on TB therapy longer than one week or living outside Lima. The study recruitment duration was between 1 January 2014 and 1 January 2015.

### Procedure

CSF was obtained during standard-of-care lumbar puncture. For the CSF collected, 6 ml was used for Ogawa modified culture and smear, 2 ml for MGIT processing and 2 ml for GeneXpert; the remainder was sent to the microbiology and biochemistry labs for additional routine testing.

#### Ogawa-modified culture

Cultures were performed at the HNDM laboratory using standard methods in accordance with the National Peruvian Guidelines. The CSF was stored refrigerated, except for 2 ml each CSF sample that was immediately centrifuged for 5 minutes. Two tubes of acidified Ogawa medium were inoculated, each with 2 drops (0.1 ml) from the sediment. If there was contamination of both of these initial cultures, then an additional 2 ml of the stored refrigerated CSF was then centrifuged. The sediment was decontaminated by the addition of 1 ml of sterile 4% sodium hydroxide solution (NaOH). After which two tubes of acidified Ogawa mediums were inoculated, each with 2 drops (0.1 ml) from the sediment. Cultures were incubated for 60 days.

#### Ziehl-Neelsen (ZN) smear

ZN smears were performed using standard methods in accordance with the National Peruvian Guidelines at the HNDM laboratory. Two drops (0.1 ml) of CSF (from the remaining 2 ml CSF centrifuged sample used for the Ogawa modified culture) was smeared on a glass microscope slide and dried. The smear was examined for a minimum of 100 fields (approximately 5 minutes) using high-power oil immersion microscopy with a 1,000x objective. Detection of a minimum of one acid-fast bacillus (AFB) was considered to be a positive result.

#### GeneXpert MTB/RIF

2 ml of CSF was processed by the Innovation for Health and Development (IFHAD) laboratory at the Universidad Peruana Cayetano Heredia, following the manufacturer’s instructions. It was first centrifuged at 3000 *g* for 15 minutes and then the pellet was re-suspended in phosphate buffered saline to a final volume of 0.5 ml. If no pellet sediment was visible after centrifuging, then the supernatant was discarded and the contents of the lowest 0.5 ml in the centrifuge tube were used as the pellet. The re-suspended sediment sample was then diluted using the 1.5 ml GeneXpert MTB/RIF sample reagent provided. The 2 ml of CSF-reagent mixture was vortexed for 30 seconds to ensure all bacteria had been re-suspended. Following the manufacturer’s instructions, the sample was left at room temperature for 15 minutes; at 10 minutes, the sample was shaken by hand 10–20 times and left to stand for the remaining 5 minutes. All of the solution was then transferred to the GeneXpert cartridge and loaded onto the GeneXpert equipment for analysis.

#### MGIT 960 culture

2 ml of CSF was centrifuged at 3000 *g* for 15 minutes. After centrifuging, the supernatant was discarded and the precipitate re-suspended in 1 ml buffer. The concentrated mixture was distributed: 0.2 ml to the Lowenstein-Jensen (LJ) tube (see below), 0.1 ml to inoculate a ZN smear, and 0.5 ml to inoculate an MGIT tube containing 0.8 ml of MGIT Growth Supplement “OADC” + PANTA antibiotic mixture (Polymyxin B, Amphotericin B, Nalidixic Acid, Trimethoprim, Azlocillin), according to the manufacturer’s protocol (Becton Dickinson, Waltham, MA USA). MGIT tubes were then incubated for a period of 42 days and automatically identified as positive, negative or contaminated by the MGIT equipment.

The decontamination step originally included in the MGIT’s manufacturer protocol was omitted for both Lowenstein-Jensen (LJ) and MGIT cultures, as is common practice because CSF is considered to be usually sterile.

#### Lowenstein-Jensen (LJ) culture

As described in the ‘MGIT 960’ section above, 0.2 ml of the concentrated CSF were inoculated onto an LJ tube. This was incubated for 56 days. Results were reported as positive, negative, or contaminated.

#### Drug resistance

Positive samples for *M*. *tuberculosis* were tested for resistance to the first-line drugs used for TB treatment. Resistance to one first-line anti-TB drug is termed “mono-resistance”. Multi-drug resistance (MDR) is defined as bacteria resistant to treatment with at least isoniazid and rifampicin. However, detection of resistance to rifampicin is considered a reliable proxy for MDR-TB and therefore in this setting causes second-line drug therapy to be initiated [14]. Therefore, samples with rifampicin-resistant TB were initially managed as MDR-TB.

Positive cultures (MGIT and LJ) were then tested for resistance to rifampicin, isoniazid, pyrazinamide, ethambutol, and streptomycin using indirect MGIT testing according to the manufacturer’s instructions. Positive Ogawa cultures underwent proportions method. GeneXpert samples tested for resistance to rifampicin only.

#### Other investigations

Routine testing included CSF: biochemistry, adenosine deaminase (ADA) level, cell count, culture and gram stain smear for meningitis. Routine testing included blood: HIV rapid test, CD4 cell count and viral load for HIV/AIDS; venereal disease research laboratory (VDRL) and rapid plasma regain (RPR) for syphilis; enzyme-linked immunosorbent assay (ELISA) for human T-lymphotropic virus (HTLV); and both India ink stain and latex agglutination test (LAT) for cryptococcal infection.

### Diagnostic classification

Patients suspected of having TBM were categorized into definite, probable, possible, and not TBM as described by Marais et al [4] and depicted in Table 1.

**Table 1:** Case definitions used in establishing a TBM diagnosis.

Category	Criterion
**(a) Clinical meningitis criteria**	Neurological symptoms such as altered consciousness, sensation or strength, seizure or dysphasia with one or more of the following: fever, headache, neck rigidity, CSF pleocytosis (> 5 leukocytes/μl), or brain imaging suggestive of meningitis
**(b) Tuberculous meningitis CSF characteristics**	Protein >40 mg/dl Glucose <40 mg/dl Leukocytes >5 white blood cells /μl ADA > 9 U/L
**(c) Tuberculous meningitis definitions**	
(c.1) Not TBM	Alternative diagnosis established, without a laboratory proven diagnosis of tuberculous meningitis from the CSF.
(c.2) TBM diagnosis	Clinical suspicion (e.g. prior TB history, cerebral imaging results) combined with abnormal CSF consistent with TBM and exclusion of other potential etiologies, causing the patient’s clinician to commence treatment for TBM.
(c.2.1) Definite TBM	Either AFB seen in the CSF smear; or *M*. *tuberculosis* cultured from the CSF; or a CSF positive GeneXpert MTB/RIF result.
(c.2.2) Probable TBM*	No AFB seen in the CSF smear; no *M*. *tuberculosis* cultured from the CSF; no CSF positive GeneXpert MTB/RIF result. May also have a positive *M*. *tuberculosis* smear and/or culture or PCR result from samples taken outside the CNS. *PLUS* ≥ 10 points on the diagnostic criteria scale (when cerebral imaging is not available) or ≥12 points (when cerebral imaging is available) with at least two points coming from either CSF or cerebral imaging criteria
(c.2.3) Possible TBM*	No AFB seen in the CSF smear; no *M*. *tuberculosis* cultured from the CSF; no CSF positive GeneXpert MTB/RIF result. May also have a positive *M*. *tuberculosis* smear and/or culture or PCR result from samples taken outside the CNS. *PLUS* A score of 6–9 points (when cerebral imaging is not available) or 6–11 points (when cerebral imaging is available)

*follows the scoring system based on the consensus uniform clinical case definition found in Marais et al [4].

As such, patients assigned to a definite TBM category required a positive *M*. *tuberculosis* smear, culture or PCR GeneXpert MTB/RIF CSF result. Patients with an unconfirmed TBM diagnosis required abnormal CSF values consistent with TBM and exclusion of other potential etiologies. Further classification of unconfirmed TBM cases into either probable or possible TBM categories were based on a scoring system outlined in the consensus uniform case definition [4].

### Statistical analysis

All statistical analyses were calculated using STATA (StataCorp, version 13.1, College Station, TX). A *p* value < 0.05 was considered to be statistically significant. Categorical variables were compared by McNemar’s test. Additionally, a laboratory diagnostic test performance analysis was conducted comparing GeneXpert MTB/RIF to culture.

## Results

Eighty patients presenting to the Hospital Nacional Dos de Mayo with suspected TBM between January and December 2014 were considered for enrollment in this study, of whom 43 patients were excluded: under 18 years old (*n* = 1), contraindication to perform an LP (*n* = 1), neurological findings on CT imaging that suggested an alternate condition other than meningitis (*n* = 13), previous history of a neurological disorder that precluded clinical evaluation (*n* = 2), TB therapy longer than one week (*n* = 3), lived outside of Lima (*n* = 3), did not sign an informed consent form (*n* = 11). Nine patients had a combination of one of the aforementioned exclusions: neurological findings on CT imaging that suggested an alternate condition + previous history of a neurological disorder that precluded clinical evaluation (*n* = 2); neurological findings on CT imaging that suggested an alternate condition + TB therapy longer than one week (*n* = 1); neurological findings on CT imaging that suggested an alternate condition + lived outside of Lima (*n* = 1); previous history of a neurological disorder that precluded clinical evaluation + lived outside of Lima (*n* = 2); under 18 years old + TB therapy longer than one week + lived outside of Lima (*n* = 1); neurological findings on CT imaging that suggested an alternate condition + TB therapy longer than one week + lived outside of Lima (*n* = 1); neurological findings on CT imaging that suggested an alternate condition + previous history of a neurological disorder that precluded clinical evaluation + TB therapy longer than one week (*n* = 1). Thus 37 patients were enrolled in the study.

**Fig 1:**
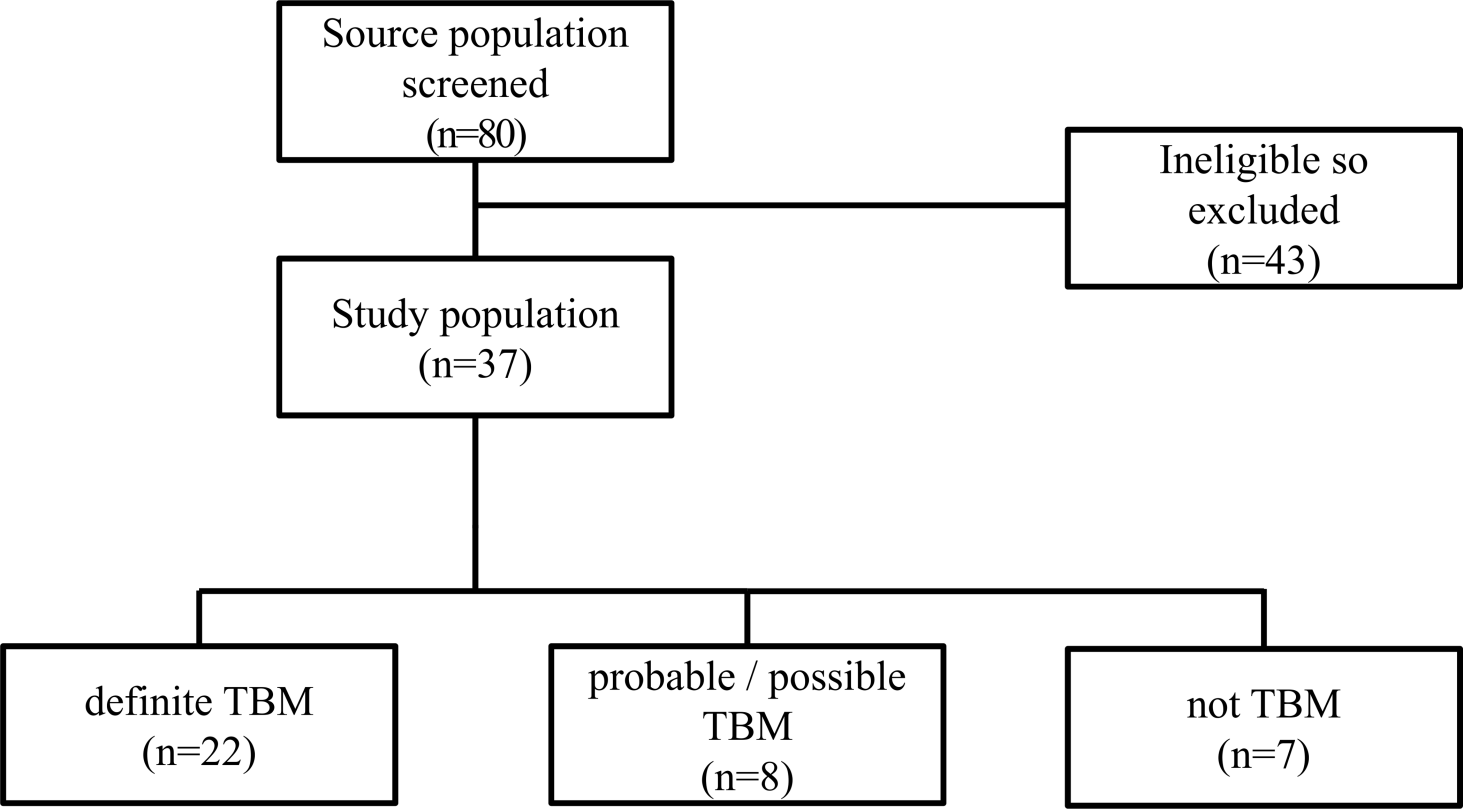
Study flowchart.

Of the 37 patients included in the analysis, 7 were classified as not having TBM: cryptococcosis (*n* = 3); toxoplasmosis (*n* = 2); psychiatric disorder (*n* = 1); and ischemic cerebrovascular disease (*n* = 1). Eight were classified as having definite TBM (*n* = 8), while the remaining 22 were classified as either probable TBM (*n* = 11) or possible TBM (*n* = 11) as their samples lacked concordance between a positive culture test and a diagnosis (Fig 1).

All enrolled patients were tested for HIV. 62% (23/37) patients were HIV co-infected. Of the patients with clinically-diagnosed TBM, 63% (19/30) were HIV co-infected. Patients’ demographic characteristics are shown in Table 2.

**Table 2:** Demographic characteristics of all study patients (patients with suspected TBM, n = 37).

Demographic characteristics	All patients (n = 37)	n (%)
Sex	Male	27 (73)
	Female	10 (27)
Age-years	18–30	10 (27)
	31–45	14 (38)
	46–60	9 (24)
	>60	4 (11)
Previous TB history	Yes	11 (30)
	No	26 (70)
HIV status	Positive	23 (62)
	Negative	14 (38)
BMRC at admission	Stage I	9 (24)
	Stage II/III	28 (76)
Duration between symptoms and admission (d)	16 ± 13 (1–60)	
Outcome	Alive	27 (73)
	Died	10 (27)

### Diagnostic test performance in patients with definite TBM

Because definite TB was defined by positivity in any microbiological test, all microbiological tests had 100% specificity.

**Fig 2:**
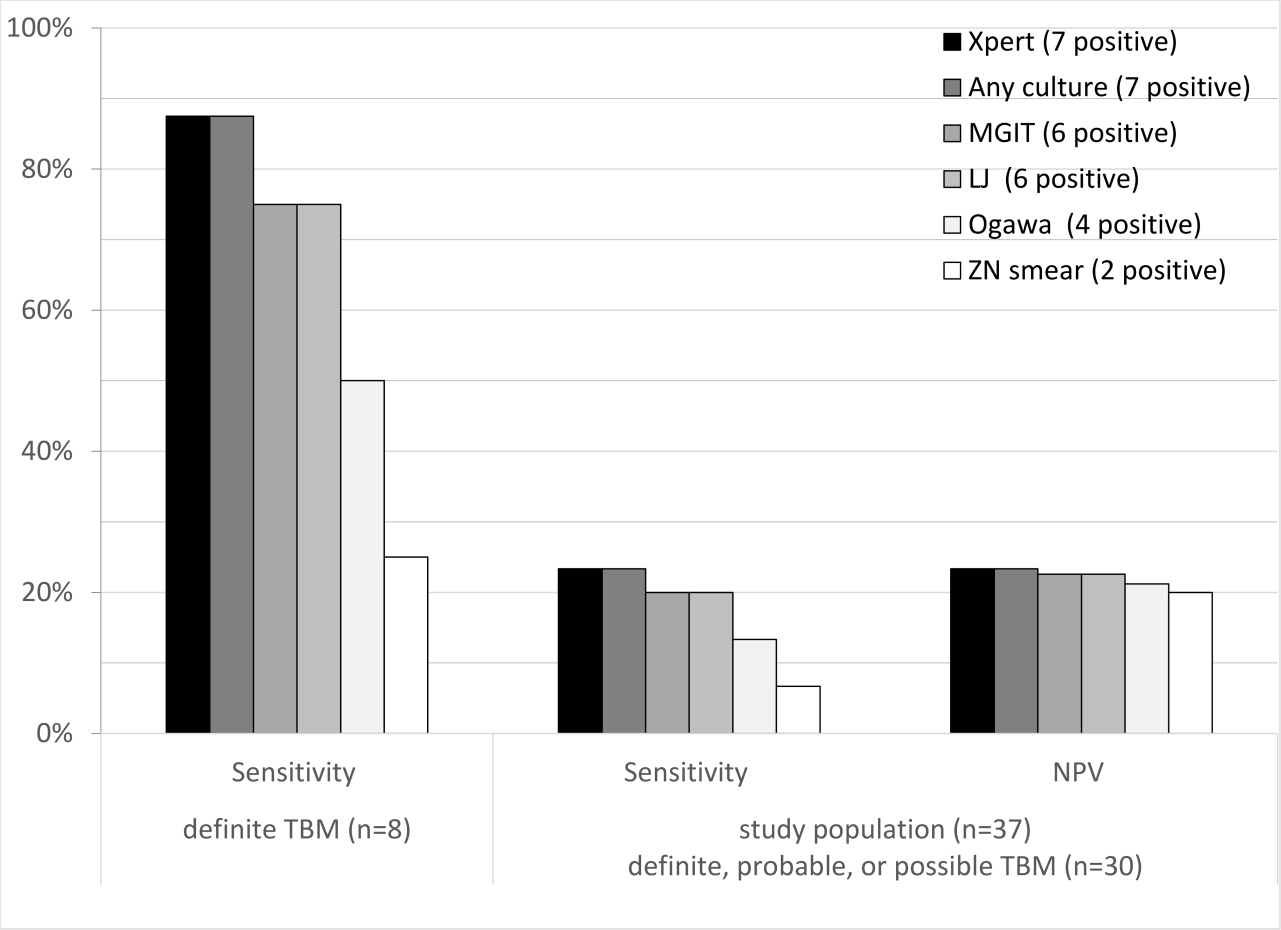
Performance of laboratory tests in definite, probable, and possible TBM diagnosis. * Values in brackets are 95% confidence intervals.

Fig 2 shows the performance of diagnostic tests including GeneXpert for the patients with a definite TBM diagnosis. Considering only these 8 patients with definite TBM: diagnostic sensitivity was 88% (7/8, 95%CI: 47–100%) for GeneXpert; 75% (6/8, 95%CI: 35–97%) for MGIT culture or LJ culture; 50% (4/8, 95%CI 16–84) for Ogawa culture and 25% (2/8, 95%CI: 3.2–65%) for microscopy.

**Fig 3:**
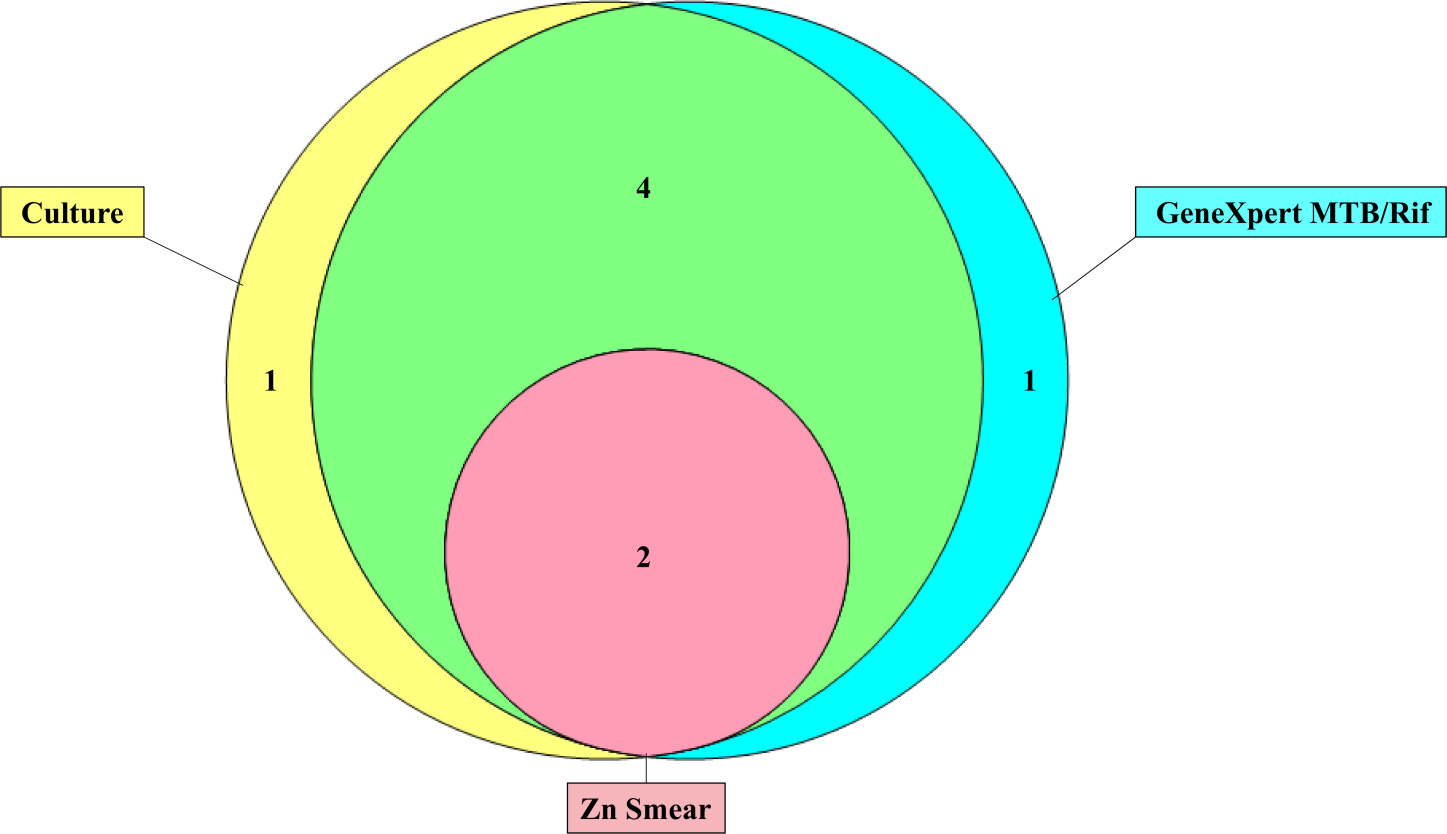
Venn diagram showing positivity by GeneXpert, culture, and ZN smear microscopy using CSF samples in patients with definite TBM (n = 8).

Fig 3 summarizes the performance of the laboratory tests used for a TBM diagnosis. One patient was positive by all five methods, three patients were positive by four methods, one patient was positive by three methods, two patients were positive by two methods, and one patient was positive by only one method. Seven patients had one or more positive laboratory test results. Specifically, one patient was positive only by GeneXpert, one patient was positive only by LJ. Ogawa culture and microscopy did not provide any additional diagnoses that were not detected by MGIT, LJ or GeneXpert.

Contamination was reported for 1 (2.7%; n = 37) MGIT culture, 1 (2.7%; n = 37) LJ culture, and none for Ogawa culture.

### Diagnostic test performance in patients with a clinical TBM

Among patients with a clinical diagnosis of TBM (definite, probable, and possible TBM), CSF culture was positive for *M*. *tuberculosis* in 23% (7/30) of patients. Among HIV co-infected patients, CSF culture was positive for *M*. *tuberculosis* in 26% (5/19).

Considering these 30 patients with a clinical diagnosis of TBM: diagnostic sensitivity was 23% (7/30, 95%CI: 9.9–42%) for GeneXpert; was the same 23% (7/30, 95%CI: 9.9–42%) for all culture results combined; considerably greater than 7% (2/30, 95%CI: 0.82–22%) for microscopy; whereas all microbiological tests had poor negative predictive values (20–23%).

### GeneXpert test performance against the gold standard (culture and smear) in patients with a clinical diagnosis of TBM

Table 3 shows the performance of specifically the GeneXpert against the gold standard (culture and smear). The GeneXpert had a sensitivity of 86% (6/7, 95% confidence interval, CI: 42–100) and 97% specificity (29/30, 95% CI: 83–100) against culture. There was no statistically significant difference between these two tests, but the sample size was small. The negative predictive value (NPV) of the GeneXpert was 97% when compared against a negative CSF culture.

**Table 3:** Diagnostic performance of GeneXpert against culture and smear as the reference standard.

	GenXpert			
	Sensitivity (95% CI)	Specificity (95% CI)	PPV (95% CI)	NPV (95% CI)
**Culture combined**^a^	86% (42–100)	97% (83–100)	86% (46–98)	97% (83–99)
MGIT	83% (36–100)	93% (78–100)	57% (25–84)	98% (90–100)
LJ	83% (36–100)	93% (78–100)	57% (25–84)	98% (90–100)
Ogawa	100% (40–100)	91% (76–99)	53% (28–77)	100% (86–100)
ZN smear	100% (16–100)	86% (70–96)	42% (18–59)	100% (80–100)

^a^Culture combined is comprised of Ogawa modified, MGIT, and LJ results.

### Test speed

**Fig 4:**
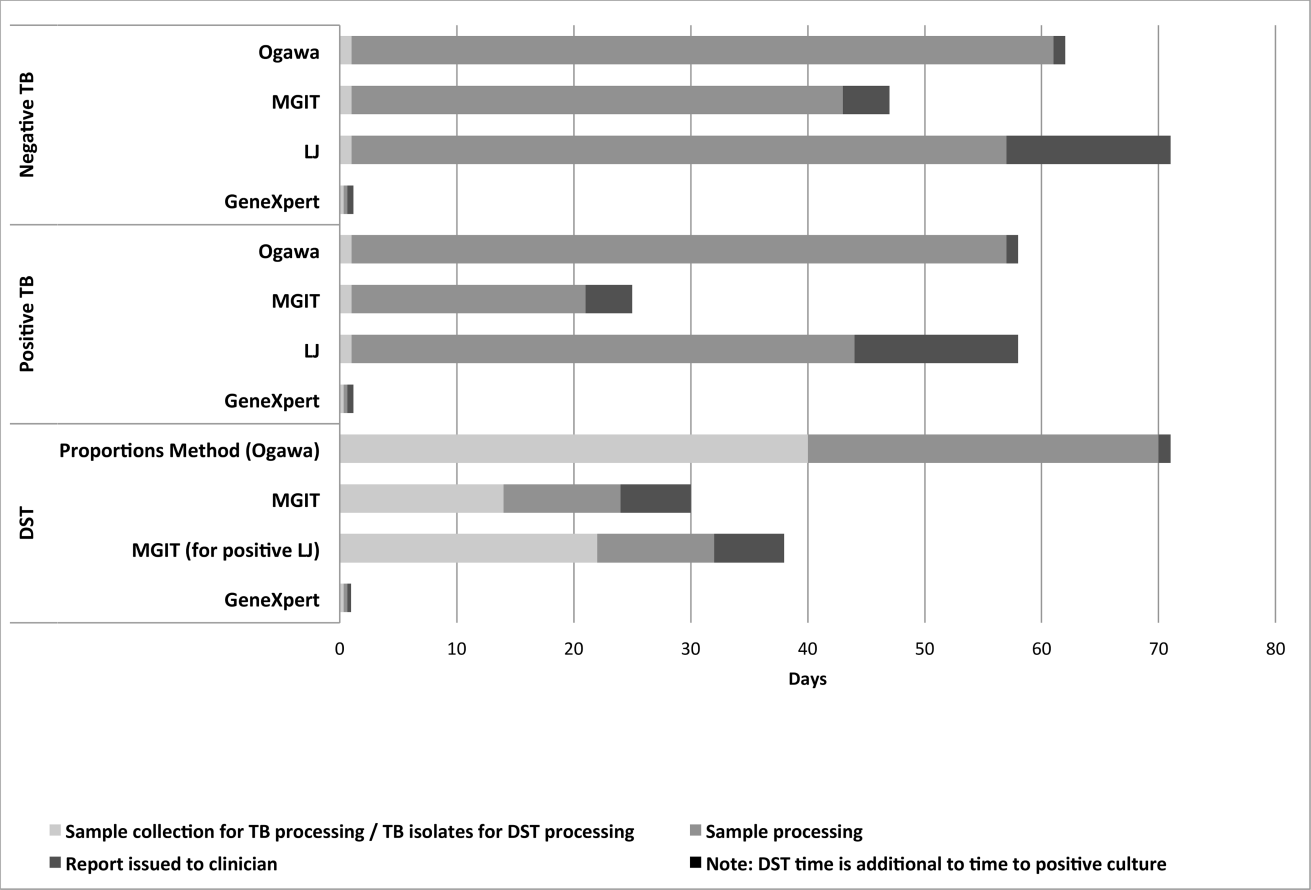
Time to results for *M*.*tuberculosis* detection and drug sensitivity testing (DST).

Fig 4 shows the number of days from sample collection until negative results, positive TB detection and TB drug-susceptibility testing results. GeneXpert and microscopy provided same-day results, whereas culture took 20–56 days. GeneXpert provided same-day rifampicin-susceptibility results, whereas culture-based testing took 32–71 days.

GeneXpert provided same-day rifampicin-susceptibility results, whereas MGIT and LJ cultures took a median of 10.5 days. Strains obtained by Ogawa culture underwent phenotypic DST at the national TB reference lab. However, these positive strains were re-tested using LJ culture prior to using the proportions method—assay times of approximately 40–45 days, and run in batches (Fig 4).

GeneXpert results, including rifampicin DST, were available usually within 2 hours of the initiation of sample processing; smear results were available within 24 hours. The median time to positivity was 40.5 days (IQR, 20.75 to 50.75 days; *n* = 7) for combined culture, and for negativity 56 days (IQR, 42 to 59 days; *n* = 30). The median time to positivity was 61 days (IQR, 55.5 to 61 days; *n* = 4) for Ogawa; 18.5 days (IQR, 11.75 to 22.25 days; *n* = 6) for MGIT; and 47 days (IQR, 39 to 49 days; *n* = 6) for LJ. The median time to negative results was 60 days (IQR, 59 to 61 days; *n* = 33) for Ogawa; MGIT tubes were incubated for a period of 42 days before they would be considered negative (*n* = 30); LJ tubes were incubated for a period of 56 days before they would be considered negative (*n* = 30).

### Drug-susceptibility testing

Drug-susceptibility testing results are shown in S1 Table. 38% (3/8, 95%CI: 8.5–76%) of patients with definite TBM with data had evidence of drug-resistant TB, but 73% (22/30) of patients with a clinical diagnosis of TBM had no drug-susceptibility results available. All 3 patients with evidence of drug-resistance were co-infected with HIV. There was insufficient data for statistical analysis of the DST accuracy of GeneXpert or the other tests used.

## Discussion

Recent recommendations from the WHO suggest that the GeneXpert be used as the initial diagnostic test in adults suspected of having MDR-TB or HIV-associated TB and for CSF specimens from patients suspected of having TB meningitis over conventional microscopy and culture [7, 8]. However, these recommendations come as a result of very low-quality evidence, so additional studies are needed [7, 8]. An estimated 58% of morbidity cases of TB and 83% morbidity cases of MDR-TB in Peru come from Lima province [15]. Two-thirds of Peru’s HIV reported cases, 66.5 percent are located in Lima, according to the Peruvian Ministry of Health [15, 16]. But the effectiveness of the GeneXpert for the diagnosis of TBM has yet to be evaluated in Peru, a country where TB and MDR-TB are highly prevalent.

The GeneXpert had identical sensitivity compared with the combined results of several CSF culture tests for detecting *M*. *tuberculosis*. The one GeneXpert-positive but culture-negative case could have been as a result of false-positive PCR caused by cross-contamination, although this seems unlikely due to the closed reaction chamber of the GeneXpert causing high specificity. Furthermore, this patient commenced empiric treatment for TBM before the microbiological results were known because TBM was considered to be clinically highly likely. Additionally, strict measures were taken by the processing laboratory to prevent cross-contamination. So this GeneXpert-positive culture-negative result is likely to have been a true positive, which culture and smear failed to identify. Additionally, there was one culture-positive case whose GeneXpert result was negative. This patient was smear-negative, HIV-positive, and had no prior history of TB; all CSF values, clinical and radiological criteria were consistent with a TBM diagnosis. The reason for this false-negative GeneXpert result may be due to a very low bacillary count in the CSF sample. The GeneXpert provides a semi-quantitative estimate of the concentration of bacilli present in a clinical sample; all 7 GeneXpert-positive samples were scored as ‘low’ and ‘very low’ suggesting a limited number of bacilli in CSF samples. Another explanation might be that the GeneXpert has a low NPV. As recommended by the WHO, patients suspected of having TBM who receive a negative GeneXpert result should undergo further diagnostic studies [7].

Concerns have been raised about the low NPV of the GeneXpert [12, 17]. In the present study, of those whose GeneXpert results in CSF were negative, the proportion of those who did not have TBM was 97% when compared against a microbiological reference standard (CSF culture and smear). This result is higher than the NPV of 84% calculated by Boyles and Thwaites based on the WHO analysis of 6 studies [7, 12]. However, when instead considering all patients treated for TBM, the NPV were remarkably low at 20–23% for all laboratory tests, including GeneXpert. Thus, a negative GeneXpert test does not rule out a TBM diagnosis.

For purposes of study analysis and future cross-comparison with other cohorts, a uniform case definition based on the Marais et al [4] TBM case criteria was applied to our research data identifying 8 cases as definite TBM, 11 as probable TBM, 11 as possible, and 7 as not TBM. It is important to point out that all 37 patients with the exception of 2 did not have cerebral imaging results, none had imaging outside the CNS performed, and none had a commercial *M*.*tuberculosis* NAAT from extra-neural specimen performed. Having had these criteria evaluated may have changed the category for some of the possible TBM cases to the probable TBM category, yet they would have been treated the same, irrespectively. The lack of availability or access to some of these medical imaging tests highlight the limitations of this diagnostic score, particularly in some resource-limited countries, like Peru.

Determining the optimal CSF volume to use and processing steps to take in order to maximize sensitivity when using the GeneXpert is another issue that requires further research. At the time our study was conducted (2014), Vadwai et al [13] had previously shown a 33% sensitivity of GeneXpert against CSF *M*.*tuberculosis* culture and 29% against a clinical reference standard (CRS). It was suggested that concentration by centrifugation and then using the pellet for processing might increase the sensitivity. In light of these findings, our study used a standard 2 ml of CSF including concentration of the sample resulting in a GeneXpert sensitivity of 88% against culture yet only 23% against a CRS. Recent publications using various volumes and concentration methods tended to demonstrate somewhat higher sensitivity results when comparing GeneXpert against culture [7, 8, 9, 10, 18, 19]. A prospective study by Patel et al in South Africa found higher sensitivity when using 3 mL of centrifuged versus 1 mL of uncentrifuged samples (82% vs 47%) [18]. A study in Vietnam reported 59% sensitivity using 2 mL or less of centrifuged CSF [10]. A more recent study in Uganda found higher sensitivity when centrifuging CSF prior to using GeneXpert [9]. It also recommended collecting a much larger volume of CSF (6–10 mL) to improve the diagnostic yield. We recommend that in future studies, less (or no) CSF is used for Ogawa culture because this technique was insensitive and instead a larger volume is centrifuge-concentrated for the more rapid and sensitive GeneXpert technique. We also recommend that if only small volumes of CSF are available for GeneXpert then the whole volume may be tested directly in the GeneXpert cartridge without dilution in the sample reagent [9].

Overall, the performance of the GeneXpert is noteworthy. The GeneXpert greatly accelerated the time-to-results with a median time of less than one day compared to 61 days using Ogawa modified culture, 19 days for MGIT, 47 days for LJ culture, and 1 day for ZN smear. Through use of GeneXpert, patients can attain an earlier diagnosis of TBM and detection of drug resistance; thereby allowing for a faster and more effective treatment, as well as potentially a higher likelihood of improved outcome, survival, and decreased neurological sequelae.

A limitation to our study was a small sample size, resulting in an inability to determine the diagnostic accuracy among subgroups (e.g. HIV status). A possible source of bias may have been introduced in that there was a lack of negative controls in the Ogawa modified cultures performed in-hospital. A formal validating scoring system to assess the clinical likelihood of TBM is lacking in the clinical practice of Peruvian hospitals. Although clinical and CSF laboratory criteria were used to determine whether clinicians chose to initiate treatment for TBM, the decision was subjective as no true score or value was assigned to each criteria. A scoring system such as the clinical algorithm outlined in the study by Marais et al [4] and that was used by us for study calculations would standardize the diagnosis of patients suspected of having TBM, particularly in those whose culture and/or smear results are negative, thereby improving patient management. It is also important to note that the GeneXpert results were communicated to clinicians but were not yet included in Peruvian policies thereby we are uncertain of the clinical impact at that time.

Our study is unique in that it is the first to report the use of GeneXpert on TBM in Latin America. Despite our small sample size, our results further support findings of other “larger” studies such as in Vietnam. This study highlights the urgent need to standardize clinical and laboratory practice for patients with TBM. This would in turn provide a more accurate measure of evaluation, as well as aid in assessment of severity and prognosis. Continued efforts to help develop an improved GeneXpert protocol for the standardization of CSF volume collection and processing methods are needed. The results of this study support the use of GeneXpert as a first line diagnostic tool for patients with suspected TBM, preferable to culture. The GeneXpert MTB/Rif assay provides faster results than any culture method, concurrent rapid detection of rifampicin-resistant *M*. *tuberculosis* strains, and optimal sensitivity compared with traditional culture-based testing.

## Supporting information

S1 Table DST results among patients with definite TBM. *Minimum of 9 colonies were not obtained for DST.
Abbreviations N.A. (Not Applicable), Neg (Negative), Pos (Positive).
DST for GeneXpert tests for resistance to Rifampicin, MGIT/LJ tests for resistance to Streptomycin, Isoniazid, Rifampicin, Ethambutol, and Pyrazinamide. Ogawa used Proportions Method testing for Streptomycin, Isoniazid, Rifampicin, and Ethambutol.
(PDF)
