# Supplementary material for: Evaluation of the GeneXpert MTB/RIF in patients with presumptive tuberculous meningitis
Source: PLoS One. 2018 Jun 18;13(6):e0198695. doi: 10.1371/journal.pone.0198695 (PMC6005529; doi:10.1371/journal.pone.0198695)
Supplement: S1 Table — *Minimum of 9 colonies were not obtained for DST. Abbreviations N.A. (Not Applicable), Neg (Negative), Pos (Positive). DST for GeneXpert tests for resistance to Rifampicin, MGIT/LJ tests for resistance to Streptomycin, Isoniazid, Rifampicin, Ethambutol, and Pyrazinamide. Ogawa used Proportions Method testing for Streptomycin, Isoniazid, Rifampicin, and Ethambutol. (PDF) [file pone.0198695.s001.pdf]

Sample Codes	TB Detection					DST				
	ZN Smear	Ogawa	MGIT	LJ	Xpert	GeneXpert RIF Resistant	MGIT/LJ RIF DST	Ogawa RIF DST	MGIT/LJ DST other than RIF	Ogawa DST other than RIF
**11004**	Neg	Neg	Neg	Pos	Pos	No Resistance	No Resistance	N.A.	Resistant to Streptomycin	N.A.
**11014**	Neg	Pos	Pos	Pos	Pos	No Resistance	No Resistance	N.A.*	No Resistance	N.A.*
**11018**	Neg	Neg	Pos	Pos	Neg	N.A.	No Resistance	N.A.	No Resistance	N.A.
**11019**	Neg	Neg	Neg	Neg	Pos	Resistant to Rifampicin	N.A.	N.A.	N.A.	N.A.
**11023**	Neg	Pos	Pos	Pos	Pos	Resistant to Rifampicin	No Resistance	Resistant to Rifampicin	Resistant to Isoniazid	Resistant to Streptomycin, Isoniazid
**11024**	Pos	Pos	Pos	Pos	Pos	No Resistance	No Resistance	N.A.*	No Resistance	N.A.*
**11025**	Neg	Neg	Pos	Pos	Pos	No Resistance	No Resistance	N.A.	No Resistance	N.A.
**11037**	Pos	Pos	Pos	Neg	Pos	No Resistance	No Resistance	No Resistance	No Resistance	No Resistance
